# The HIP mouse and all of its organs are completely invisible to allogeneic immune cells

**DOI:** 10.1016/j.isci.2024.111492

**Published:** 2024-11-28

**Authors:** Xiaomeng Hu, Kathy White, Ari G. Olroyd, Chenyan Wang, Carolin B. Caruso, Corie Gattis, Chi Young, Andrew J. Connolly, Tobias Deuse, Sonja Schrepfer

**Affiliations:** 1 Sana Biotechnology Inc., 1 Tower Place, South San Francisco, CA, USA; 2 Department of Pathology, University of California, San Francisco, San Francisco, CA 94143, USA; 3 Department of Surgery, Division of Cardiothoracic Surgery, Transplant and Stem Cell Immunobiology (TSI)-Lab, University of California, San Francisco, San Francisco, CA, USA

**Keywords:** Natural sciences, Biological sciences, Immunology, Immune response

## Abstract

Hypoimmune (HIP) allogeneic cell therapeutics hold the promise to allow off-the-shelf treatments for a broad patient population. Our HIP approach includes the depletion of major histocompatibility complex (MHC) class I and II molecules and the overexpression of Cd47. Here, we report the engineering of HIP mice that stably exhibit the HIP phenotype in all cell types. Parabiosis experiments were designed to broadly assess immune evasiveness of all HIP blood cells in fully allogeneic BALB/c mice. HIP blood cells did not induce any immune response and achieved stable engraftment in BALB/c mice. Parabiosis experiments with irradiated HIP mice served as a model for full-body transplantation. There was no measurable cellular or antibody response in immunocompetent, allogeneic BALB/c parabionts. Transplantation of HIP islets into diabetic, allogeneic BALB/c mice reliably treated diabetes in all animals. Together, these data suggest that all allogeneic tissues can be HIP engineered and HIP cell therapy may be envisioned for many more indications.

## Highlights

• HIP mice with MHC I and II depletion and Cd47 overexpression are viable and healthy
• Transferred HIP blood cells achieved stable engraftment in allogeneic mice
• Primary HIP islets engraft and treat diabetes in immunocompetent allogeneic mice
• Full-body transplantation of HIP mice induces no allogeneic immune response

## Graphical abstract

**Figure:**
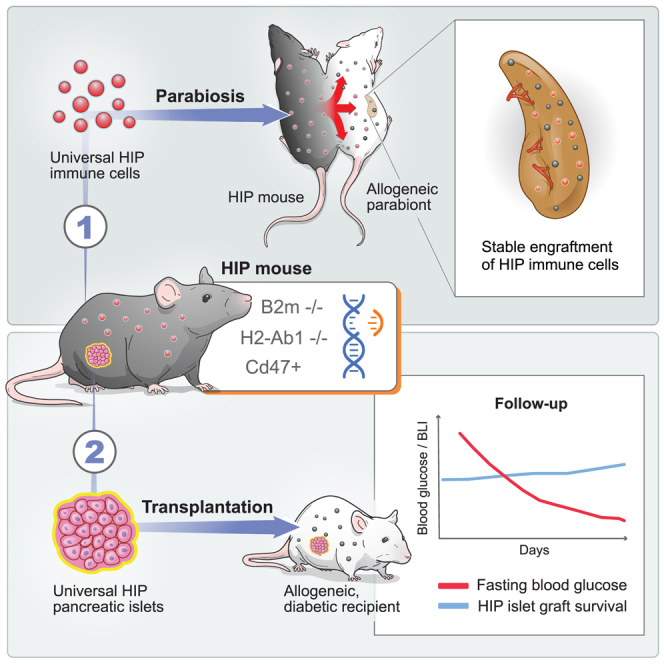

## Introduction

Successful examples of murine hypoimmune (HIP)-engineered cell types that achieved long-term survival in fully allogeneic hosts include endothelial cells,^1^^,^^2^ cardiomyocytes,^1^^,^^2^ smooth muscle cells,^1^ and pancreatic β cells.^3^ However, different cell types can have different levels of immunogenicity that are affected by their expression of co-stimulatory ligands, adhesion molecules, immune checkpoint ligands, or immune-activating ligands. The aim of this study was to broadly test whether HIP engineering is sufficient for all cells, tissues, and organs to become immune evasive and circumvent any immune recognition in fully allogeneic recipients. To allow the testing of all cell types at the same time, we first generated HIP mice that exhibit the HIP phenotype of major histocompatibility complex (MHC) class I and II deficiency together with overexpression of Cd47 in all cells of the body. Then, experiments were designed that allow the overall assessment of immune activation of all blood cells or all organs simultaneously with fully allogeneic recipients. We chose different experimental parabiosis designs with or without irradiation of the HIP parabionts to allow for unbiased, broad immune analyses. We found that the entire HIP mouse was completely invisible to a fully competent, allogeneic immune system.

## Results

### The generation of HIP mice

A homozygous HIP mouse on the C57BL/6 (B6) background that lacked both MHC I and II expression and overexpressed Cd47 was generated. First, mice with knockouts in *B2m* or *H2-Ab1* were crossed to make an MHC class I and II-deficient double knockout strain^4^ (Figure S1A). A mouse overexpressing Cd47 and expressing the firefly luciferase-encoding *Luc2* was generated by targeted transgenesis into mouse embryonic stem cells, with subsequent injection of confirmed clones into blastocysts and transfer into pseudopregnant females for chimera generation (Figure S1B). HIP mice were obtained by intercrossing of both strains, and the desired female homozygous HIP offspring was selected for this study (Figure S1C). HIP mice were viable, healthy, and showed normal life expectancy. Flow cytometry was performed on blood and single-cell suspensions from multiple organs of HIP mice and confirmed the HIP phenotype across all cells and organs (Figure S2). The suitability of HIP mice to function as universal donors for allogeneic transplantation of blood cells and organs was assessed next.

### The parabiosis model

**Figure 1:**
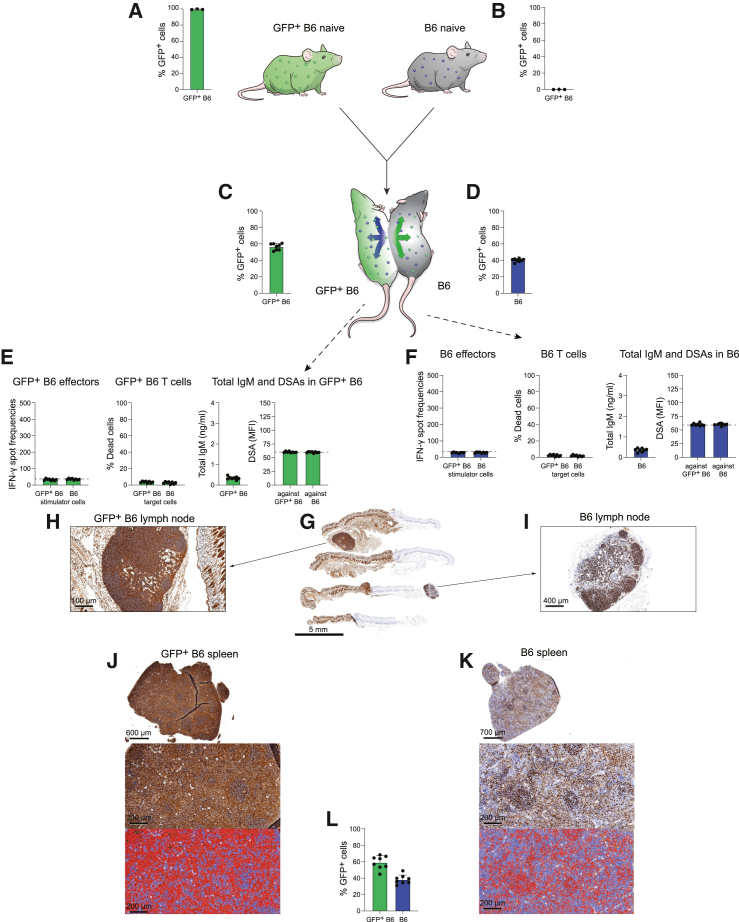
Parabiosis with two syngeneic parabionts (A and B) GFP+ B6 naive mice are depicted in green with green immune cells, and B6 naive mice are depicted in gray with blue immune cells. Virtually all blood cells in GFP+ animals were positive for GFP (A), while no GFP fluorescence was observed in blood cells of regular B6 animals (B) (mean ± SD, 3 animals per group). (C and D) After two weeks in parabiosis, immune cells from each mouse had crossed over into the other parabiont, creating an immune cell mix in the common blood circulation. The equilibrium in each mouse was skewed mildly toward an approximate 60%: 40% dominance of the own inherit blood population (mean ± SD, 8 animals per group). (E) An immune response analysis in GFP+ B6 mice showed no activation of splenocytes (Elispot) and no killing (Live-Dead assay) against either GFP+ B6 or B6. Total IgM serum levels remained in the norm, and no DSAs against either GFP+ B6 or B6 were observed (mean ± SD, 8 animals per group; the dashed lines in the Elispot and DSA graphs show the background levels of these assays). (F) An immune response analysis in B6 mice similarly showed no activation of splenocytes (Elispot) and no killing (Live-Dead assay) against either GFP+ B6 or B6. Total IgM serum levels also remained in the norm, and no DSAs against either GFP+ B6 or B6 were observed (mean ± SD, 8 animals per group; the dashed lines in the Elispot and DSA graphs show the background levels of these assays). (G) The site of the surgical connection between the parabionts was resected, cut, and histochemically stained for GFP. Four different levels are shown (one exemplary parabiosis pair is shown of 8 total, scale bar 5 mm). (H and I) GFP histochemistry staining of local lymph nodes in GFP+ B6 (H, scale bar 100 μm) and B6 (I, scale bar 400 μm) shows the composition of GFP-positive and GFP-negative immune cells (one exemplary lymph node is shown per parabiont). (J and K) The spleens were recovered from the GFP+ B6 (J, scale bar 600 μm for the top image and 200 μm for the bottom images) and B6 (K, scale bar 700 μm for the top image and 200 μm for the bottom images) parabiont, cut, and histochemically stained for GFP. Automated image analyses for brown GFP staining were performed, and the detected areas are visualized in red pseudocolor (one exemplary spleen per parabiont is shown out of 8 parabiosis pairs). (L) The percent covered area by red pseudocolor was quantified and showed an approximately 60%: 40% distribution of own inherit immune cells over those of the other parabiont (mean ± SD, 8 animals per group).

For parabiosis experiments, two animals get surgically connected at their flanks so that they share each other’s blood circulation. This allows the assessment of immune responses the mice mount against each other. Compared to adoptive transfer of immune cells through intravenous injection into a recipient, the parabiosis model generates one common circulation and thus much greater and more persistent exposure to the immune cells of the other parabiont. It further allows evaluation of the complex immune response as it affects each of the parabionts individually. After 1–2 weeks, parabiotic mice have the ability to ambulate normally. In a first syngeneic experiment, GFP+ B6 mice were connected with syngeneic B6 mice (Figures 1A and 1B). As expected, all blood cells in GFP+ B6 mice were GFP positive, and no cells in B6 were GFP positive. After 14 days in parabiosis, the blood populations were largely mixed across both mice, with minor dominance of their respective native GFP status (Figures 1C and 1D). Splenocytes were recovered from both animals, and T cells were isolated through CD3 sorting. Elispot assays showed no immune activation of either GFP+ B6 or B6 splenocytes against the other cell population, and T cell killing assays showed no cytotoxicity against the other animal’s immune cells (Figures 1E and 1F). In serum recovered from both animals, total immunoglobulin (Ig)M titers were low and no donor-specific antibodies (DSAs) against either animal were detectable (Figures 1E and 1F). Thus, in this syngeneic model, no immune response was observed. The skin and subcutaneous tissue of the surgical connection between both animals were recovered and stained for GFP expression (Figure 1G). GFP+ immune cells from GFP+ B6 mice had migrated and accumulated in naive B6 mice and vice versa (Figures 1H and 1I). This led to the dilution of brown GFP staining in GFP+ B6 mice and an enrichment of GFP staining in naive B6 mice. Similarly, in recovered spleen, there was this washout phenomenon of GFP staining in GFP+ B6 mice and the enrichment of GFP+ cells in B6 spleen (Figures 1J and 1K). Automated QuPath quantification of GFP-positive cells in spleens of both groups revealed a thorough mixing between the two mice with a similar approximately 60%–40% distribution of inherent versus transplanted immune cells (Figure 1L).

**Figure 2:**
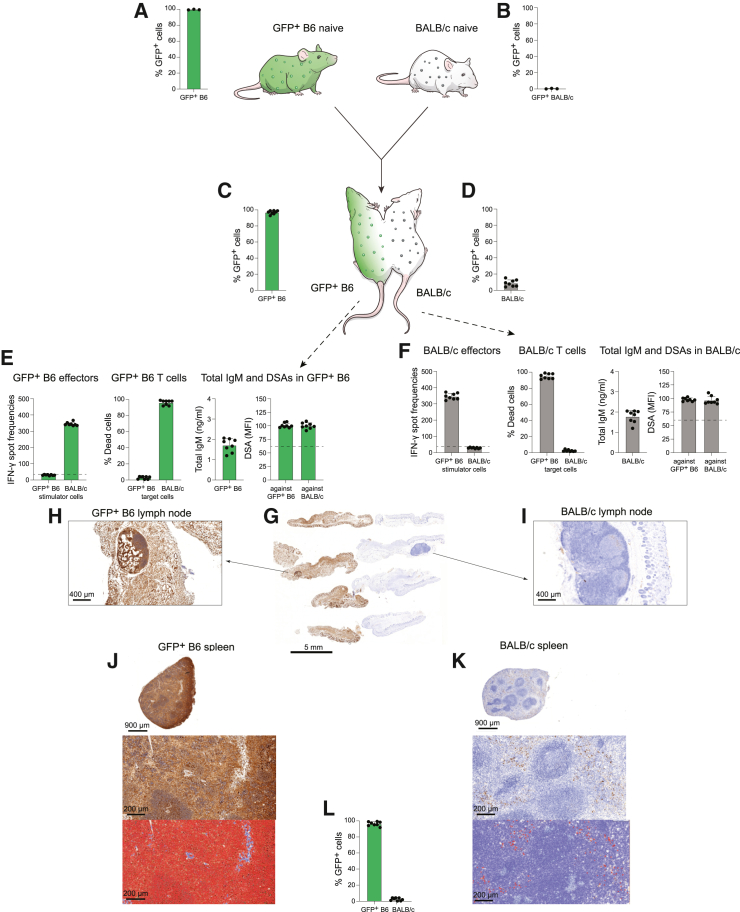
Parabiosis with two allogeneic parabionts (A and B) GFP+ B6 naive mice are depicted in green with green immune cells, and BALB/c naive mice are depicted in white with gray immune cells. Virtually all blood cells in GFP+ animals were positive for GFP (A; the same 3 animals are shown from Figure 1A), while no GFP fluorescence was observed in blood cells of regular BALB/c animals (B) (mean ± SD, 3 animals per group). (C and D) After two weeks in parabiosis, there was minimal mixing of immune cells as only very few immune cells from the other parabiont could be detected in each mouse (mean ± SD, 8 animals per group). (E) An immune response analysis in GFP+ B6 mice showed high IFN-γ spot frequencies in Elispot assays with BALB/c stimulator cells and very effective killing of BALB/c target cells by GFP+ B6 T cells in Live-Dead assays. No response against syngeneic GFP+ B6 cells was observed. Total IgM serum levels were elevated, and DSAs against both the own GFP+ B6 strain and BALB/c were detected (mean ± SD, 8 animals per group; the dashed lines in the Elispot and DSA graphs show the background levels of these assays). (F) An immune response analysis in BALB/c mice similarly showed strong activation of splenocytes (Elispot) against GFP+ B6 immune cells and effective killing of GFP+ B6 cells by BALB/c T cells in Live-Dead assays. Total IgM serum levels were also elevated, and, again, DSAs against both the own BALB/c and GFP+ B6 strain were detected (mean ± SD, 8 animals per group; the dashed lines in the Elispot and DSA graphs show the background levels of these assays). (G) The site of the surgical connection between the parabionts was resected, cut, and histochemically stained for GFP. Four different levels are shown (one exemplary parabiosis pair is shown of 8 total, scale bar 5 mm). (H and I) GFP histochemistry staining of local lymph nodes in GFP+ B6 (H) and BALB/c (I) shows the composition of GFP-positive and GFP-negative immune cells (one exemplary lymph node is shown per parabiont, scale bar 400 μm). (J and K) The spleens were recovered from the GFP+ B6 (J) and BALB/c (K) parabiont, cut, and histochemically stained for GFP. Automated image analyses for brown GFP staining were performed, and the detected areas are visualized in red pseudocolor (one exemplary spleen per parabiont is shown out of 8 parabiosis pairs, scale bar 900 μm for the top image and 200 μm for the bottom images). (L) The percent covered area by red pseudocolor was quantified and showed that almost all immune cells were of the own parabiont’s origin with very few crossed over cells from the other parabiont (mean ± SD, 8 animals per group).

The results of the allogeneic parabiosis experiment, in which GFP+ B6 mice were connected to fully allogeneic BALB/c mice, were very different (Figures 2A and 2B). After 14 days in parabiosis, we saw barely any persistence of crossed over blood cells in the respective partner animals (Figures 2C and 2D). Immune analyses showed a strong interferon (IFN)-γ response against the other mouse strain with aggressive T cell killing (Figures 2E and 2F). There was an increase in total IgM antibodies and DSAs against both animals, and such antibodies could be similarly detected in both animals. That assumes that, due to the effective mixing of blood, antibodies generated in one animal freely cross over to the other animal. In the tissue bridge between the animals and adjacent lymph nodes as well as in the spleens, we did not observe any immune cell mixing (Figures 2G–2L). The data suggest that each mouse mounts a strong immune response against the other parabiont, which prevents any persistence of blood cells that crossed over. Together, these data describe the parabiosis model and facilitate the interpretation of the subsequent parabiosis experiments with HIP mice.

### HIP mice are universal donors for blood cell products

**Figure 3:**
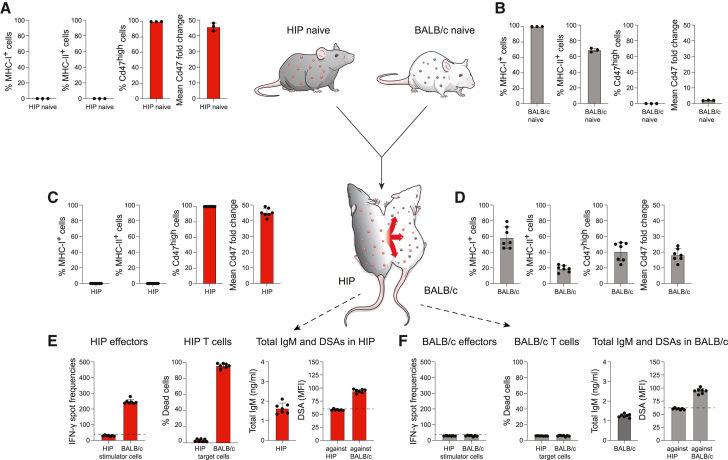
Parabiosis of HIP with allogeneic BALB/c: Testing the immunogenicity of all HIP blood cells (A) The phenotype of HIP immune cells includes a depletion of MHC class I and II and high Cd47 expression, which has an mean fluorescence intensity (MFI) approximately 45-fold higher than that of the corresponding isotype control. Mean ± SD, 3 animals. (B) BALB/c immune cells are MHC class I positive, and approximately 70% are positive for MHC class II. Endogenous Cd47 expression has an MFI approximately 2-fold higher than that of the corresponding isotype control, and BALB/c have no Cd47 high-expressing cells. Mean ± SD, 3 animals. (C) After two weeks in parabiosis, virtually all circulating immune cells in HIP parabionts still showed the HIP phenotype and close to no BALB/c immune cells could be detected (mean ± SD, 7 animals). (D) In stark contrast, approximately 40% of all circulating immune cells in BALB/c were of HIP origin, identified by MHC class I negativity and high Cd47 expression (mean ± SD, 7 animals). (E) HIP mice had developed a strong and specific immune activation against BALB/c (Elispot), and HIP T cells aggressively killed BALB/c target cells in Live-Dead assays. Total IgM serum levels were elevated compared to syngeneic (Figure 1), and DSAs only against BALB/c were detected (mean ± SD, 7 animals; the dashed lines in the Elispot and DSA graphs show the background levels of these assays). (F) BALB/c mice showed no immune activation against HIP cells (Elispot) and no killing capacity against HIP cells (Live-Dead assays). Total IgM serum levels were also elevated, and only DSAs against its own BALB/c strain were detected (mean ± SD, 7 animals; the dashed lines in the Elispot and DSA graphs show the background levels of these assays).

HIP mice were connected to fully allogeneic naive BALB/c mice in parabiosis (Figures 3A and 3B). Splenocytes from HIP mice uniformly showed the HIP phenotype of MHC class I and II deficiency and Cd47^high^ expression with a mean fluorescence intensity of approximately 45-fold over the isotype control (Figure 3A). In contrast, all BALB/c splenocytes were MHC class I positive, and approximately 70% were MHC class II expressing. Naive BALB/c mice did not have any Cd47^high^ cells, and the mean fluorescence intensity change of Cd47 on splenocytes was only approximately 2-fold over the isotype control and thus much lower than that of HIP cells (Figure 3B). Fourteen days later, the immune phenotype of splenocytes in the HIP parabiont had not changed at all, which points to the very successful rejection of all BALB/c cells that had crossed over (Figure 3C). In stark contrast, native BALB/c splenocytes in the BALB/c parabiont had decreased to around 60% of all cells with 40% having been replaced with cells of HIP origin (Figure 3D). That is similar to the mixing equilibrium previously seen in syngeneic parabionts and supports the notion that BALB/c mice accept HIP blood cells as if they were syngeneic (Figure 1D). HIP mice showed very aggressive killing of BALB/c cells and also induced BALB/c-directed DSAs as part of their effective immune response against the allogeneic parabiont (Figure 3E). The immunological acceptance and engraftment of HIP blood cells in BALB/c were enabled by the complete lack of immune recognition and immune response of BALB/c effector cells. Total IgM was increased in the common circulation and detected at the same levels in both parabiosis partners. No DSAs against HIP cells were identified, but DSAs against BALB/c were present, even in BALB/c mice (Figures 3E and 3F). These data show that no HIP blood cells that entered BALB/c induced any immune response. The HIP phenotype is thus sufficient for all blood cell type populations to fully escape immune recognition in an immunocompetent fully allogeneic recipient.

### HIP mice are universal donors for solid organ transplantation

**Figure 4:**
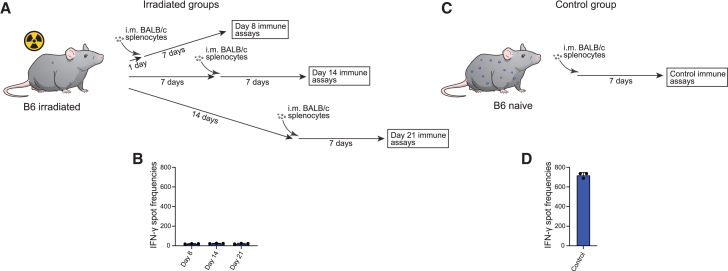
Induced immune deficiency with irradiation (A) B6 mice were irradiated with 5 Gy and subsequently immunized with intramuscular injection of allogeneic BALB/c splenocytes 1, 7, or 14 days later. Another 7 days after the immunization, the spleens were recovered and splenocytes were isolated for Elispot assays. (B) Elispot assays with the recovered B6 splenocytes and BALB/c stimulator cells did not show an increase in IFN-γ spot frequencies and thus no signs of immune activation (mean ± SD, 3 animals). (C) Naive B6 mice were intramuscularly injected with BALB/c splenocytes, and their spleens were recovered after 7 days. (D) Elispot assays showed a very strong IFN-γ response in this allogeneic control group (mean ± SD, 3 animals).

Immunocompetent HIP mice in parabiosis very effectively reject allogeneic immune cells that cross over from the connected parabiont. To enable the HIP mice to be perfused with allogeneic blood, their immune system needed to be disabled. We performed total body irradiation of B6 mice with 5 Gy and tested their ability to recognize intramuscularly injected allogeneic BALB/c splenocytes (Figure 4A). BALB/c splenocytes were injected 1, 7, or 14 days after the irradiation, and splenocytes were recovered 7 days later for Elispot assays. At no time point did we see any IFN-γ response mounted by the irradiated B6 mice, which points to effective immune ablation with this irradiation conditioning (Figure 4B). Intramuscular injections of allogeneic BALB/c splenocytes into immunocompetent B6 recipients mounted a very strong IFN-γ response with spot frequencies around 700, which is typical for a vigorous immune response in a stringent mouse transplant model (Figures 4C and 4D). This irradiation conditioning was then applied to HIP parabionts.

**Figure 5:**
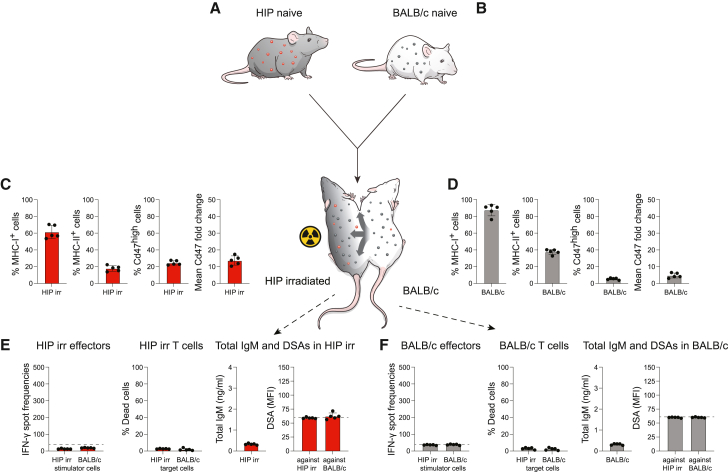
Parabiosis of irradiated HIP with allogeneic BALB/c: Testing the immunogenicity of all HIP tissues and organs (A and B) HIP mice and BALB/c mice were again joined in parabiosis, but this time the HIP mice were irradiated with 5 Gy before the surgical connection. (C) After 7 days in parabiosis, the majority of circulating immune cells in irradiated HIP mice were MHC class I positive with a minority of cells showing the inherit HIP phenotype (mean ± SD, 5 animals). Most immune cells in Hip were therefore of BALB/c origin. (D) The vast majority of immune cells in BALB/c were still MHC class I positive with only a very small fraction of Cd47 high-expressing HIP cells (mean ± SD, 5 animals). (E) The few remaining HIP immune cells in irradiated HIP mice were unable to get activated by (Elispot) or respond against BALB/c (Live-Dead assays). Total IgM serum levels remained in the normal range, and no DSAs were detected (mean ± SD, 5 animals; the dashed lines in the Elispot and DSA graphs show the background levels of these assays). (F) Despite circulating freely through irradiated HIP mice, BALB/c immune cells showed no activation against HIP cells (Elispot) and no killing capacity against HIP cells (Live-Dead assays). Total IgM serum levels were normal, and no DSAs were detected (mean ± SD, 5 animals; the dashed lines in the Elispot and DSA graphs show the background levels of these assays).

HIP mice were irradiated with 5 Gy and connected to allogeneic, immunocompetent naive BALB/c mice for 7 days (Figures 5A and 5B). Since BALB/c immune cells were able to circulate freely in HIP, this was *de facto* a full-body transplantation model including all organs at the same time. HIP splenocytes after 7 days were mainly made up of MHC class I-expressing BALB/c immune cells, with a minority of non-functional native HIP cells (Figure 5C). The immune cell composition in BALB/c spleens did not change markedly during parabiosis, which speaks to the decimation of overall irradiated HIP immune cells in the common circulation (Figure 5D). The few remaining HIP immune cells were unable to recognize or respond to allogeneic BALB/c targets as sign of the vast immune deficiency in these irradiated parabionts (Figure 5E). In this full-body transplantation model, there was no measurable immune activation by BALB/c effector cells against HIP cells, there was no increase in total IgM, and there were no measurable DSAs against HIP (Figures 5E and 5F). The data therefore suggest that the entire HIP mouse was immunologically invisible to BALB/c immune cells. HIP engineering should thus enable each individual organ to be immune protected and be transplantable into allogeneic recipients without risk for rejection.

### Pancreatic islets from HIP mice treat diabetes in allogeneic, diabetic BALB/c mice

**Figure 6:**
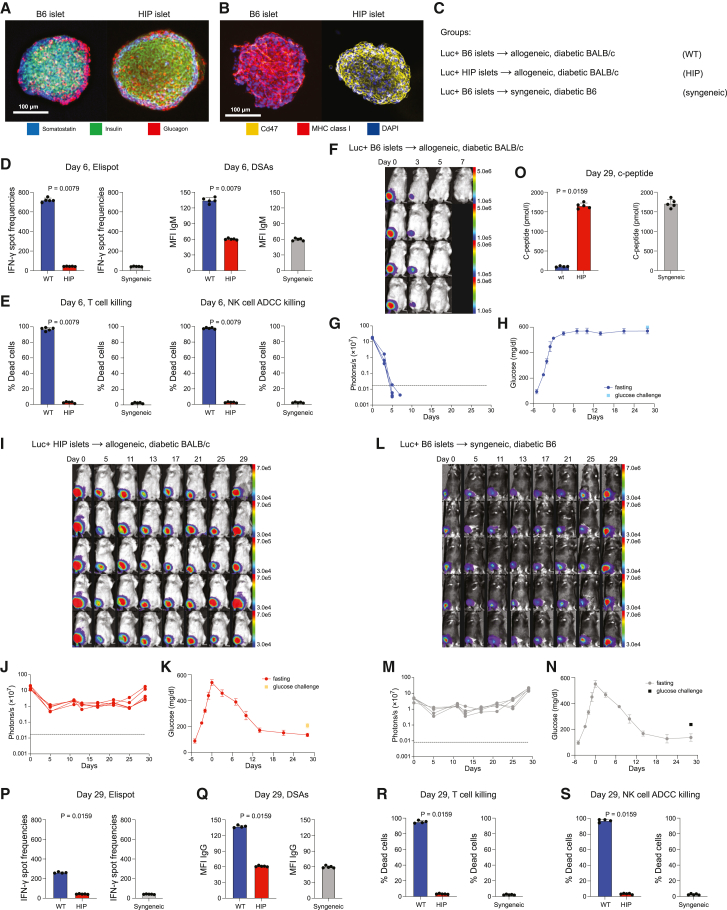
Islets from HIP mice treat diabetes in fully allogeneic, diabetic BALB/c mice (A) *In vitro* immunofluorescence stainings of B6 islets or HIP islets for somatostatin, insulin, and glucagon are shown (representative pictures of two independent experiments, scale bar 100 μm). (B) Immunofluorescence stainings for Cd47, MHC class I, and DAPI show the differences in immune phenotype between B6 and HIP islets (representative pictures of two independent experiments, scale bar 100 μm). (C) The study groups included the transplantation of B6 wild-type (WT) islets and HIP islets into allogeneic, diabetic BALB/c mice. Syngeneic transplants of B6 islets into diabetic B6 recipients served as controls. (D) Six days after islet transplantation, serum and splenocytes were recovered from the recipient mice and splenocytes were used for Elispot assays against the transplanted islet cell population. There was a strong immune activation against the allogeneic WT islets, but not the HIP islets. No immune activation was seen in the syngeneic control group (Mann-Whitney test, 5 animals per group). IgM DSAs were quantified by flow cytometry in the recovered serum. DSAs could only be detected after the transplantation of WT islets, but not HIP islets and not in the syngeneic control group (Mann-Whitney test, 5 animals per group). (E) T cells were isolated from the recovered spleens and used in cytotoxicity assays against the transplanted islet cell population. There was very effective killing of WT islets but no killing of allogeneic HIP islets or syngeneic control islets. antibody-dependent cellular cytotoxicity (ADCC) with recovered serum and allogeneic NK cells showed effective antibody-mediated killing of WT islets but not HIP or syngeneic control islets (Mann-Whitney test, 5 animals per group). (F and G) Luc+ B6 islets were transplanted into allogeneic, diabetic BALB/c mice (BLI pictures (F) and signals (G) for all 4 animals are shown) and vanished quickly. (H) Fasting blood glucose levels and 30-min glucose challenge levels showed continuation of the diabetic hyperglycemia after WT islet transplantation (mean ± SD, 4 animals). (I and J) Luc+ HIP islets were transplanted into allogeneic, diabetic BALB/c mice (BLI pictures (I) and signals (J) for all 5 animals are shown) and showed stable engraftment. (K) Fasting blood glucose levels and 30-min glucose challenge levels showed a reduction of hyperglycemia with successful treatment of diabetes after 29 days (mean ± SD, 5 animals). (L and M) Luc+ B6 islets were transplanted into syngeneic, diabetic B6 mice (BLI pictures (L) and signals (M) for all 5 animals are shown) and showed stable engraftment. (N) Fasting blood glucose levels and 30-min glucose challenge levels showed a reduction of hyperglycemia with successful treatment of diabetes after 29 days (mean ± SD, 5 animals). (O) Blood c-peptide levels were measured after 29 days. Mann-Whitney test, (mean ± SD, 4 WT animals and 5 HIP and syngeneic control animals) and showed restoration with allogeneic HIP and syngeneic B6 islet transplants. (P–S) The recipient immune response was assessed again at 29 days. The IFN-γ spot frequency was lower against WT islets (P), and DSAs had switched to the IgG isotype (Q). T cell killing (R) and NK cell ADCC (S) against WT islets were still very effective, and no killing of the other islet populations was seen (Mann-Whitney test, mean ± SD, 4 WT animals and 5 HIP and syngeneic control animals).

Pancreatic islets were chosen to test the transplantation of individual HIP organs since their survival, engraftment, and functional competence can all be quantitatively assessed. Pancreatic islets were isolated from naive B6 mice and HIP mice and showed similar composition of somatostatin-, insulin-, and glucagon-containing cell populations (Figure 6A). HIP islets were MHC class I and II negative and showed bright Cd47 staining on immune fluorescence (Figure 6B). B6 and HIP islets were dissociated and re-clustered, which allowed the transduction of B6 islet cells with firefly luciferase because they did not have inherent Luc activity. Diabetic BALB/c mice received either allogeneic wild-type (WT) B6 islets or allogeneic HIP islets. Syngeneic B6 islets into diabetic B6 mice served as controls (Figure 6C). The recipient immune responses against the transplanted allogeneic WT and HIP islets were assessed after 6 days, with the syngeneic group establishing baseline levels. Elispot assays showed a very strong IFN-γ response against WT but no response against HIP (Figure 6D). Similarly, BALB/c recipients mounted an IgM antibody response against WT islets but not HIP islets. The increased cellular and antibody responses corresponded to increased WT islet T cell killing by BALB/c T cells and increased antibody-dependent cellular cytotoxicity mediated by BALB/c serum and natural killer (NK) cells (Figure 6E). No cellular or antibody-mediated killing of HIP islets was observed. Three hundred Luc+ WT B6 islets were transplanted via intramuscular injections into allogeneic, diabetic BALB/c recipients and were followed by longitudinal bioluminescence imaging (BLI) imaging (Figure 6F). The Luc signal dropped rapidly, and all grafts were rejected within a week of transplantation (Figure 6G). Fasting blood glucose was monitored until 29 days after islet transplantation (Figure 6H). Six days after the start of the streptozotocin (STZ) treatment, when the WT islets were injected, mean fasting glucose was above 400 mg/dL. The transplanted WT islets had no effect on blood glucose, and all animals remained diabetic. Next, Luc+ HIP islets were injected into allogeneic, diabetic BALB/c mice (Figure 6I). The BLI signal dropped from the time of transplantation to day 5 but then recovered and maintained stable for the remainder of the study (Figure 6J). Fasting blood glucose was similarly elevated at the time of HIP islet transplantation but showed a steady decline thereafter (Figure 6K). After 14 days, fasting blood glucose remained stably under 200 mg/dL. In all mice of this group, diabetes was successfully treated. Then, syngeneic B6 islets were injected into diabetic B6 recipients (Figure 6L). The course of the BLI signal in this syngeneic control group was almost identical to that of the allogeneic HIP group (Figures 6M and 6J). Also, all animals were rescued from diabetes at a pace very similar to that seen with allogeneic HIP islets (Figure 6N). On day 29, spleens and serum were recovered in all animals. In the WT group, c-peptide levels were largely diminished and reflected the diabetic state of the animals (Figure 6O). C-peptide had recovered both with allogeneic HIP and syngeneic B6 islets. A thorough immune analysis was performed. Elispot assays still showed elevated spot frequencies in the WT group, although the overall number had come down compared to the earlier 6-day time point (Figure 6P). Animals in the HIP group still did not show any IFN-γ response. An isotype switch had taken place in the WT group, and we could now detect elevated IgG DSAs against the grafted islets (Figure 6Q). There were no IgG DSAs detectable in the HIP group. We found BALB/c T cell killing and antibody-mediated NK cell killing of WT islets but no cytotoxicity against HIP grafts (Figures 6R and 6S). Overall, islets from HIP mice could be transplanted in fully allogeneic, diabetic mice and fully escaped immune recognition, showed successful engraftment, and treated diabetes in all animals.

## Discussion

We know from the field of transplantation that some organs evoke inherently stronger or weaker rejection responses.^5^ In mice, kidney and liver transplants across MHC barriers induce a weak rejection response, and spontaneous tolerance is regularly observed in the absence of immunosuppression.^6^^,^^7^ In contrast, heart, lung, and small intestine allografts induce a more vigorous immune response, and achieving transplantation tolerance is difficult.^8^ Therefore, different cell types may require different HIP editing strategies. Besides our HIP engineering concept, other groups have come up with different editing approaches^9^^,^^10^^,^^11^ based on different hypotheses, and the suitability of HIP for all organs and tissues remained speculative.

Parabiosis models can be used in a variety of different ways to assess different specific aspects of immunogenicity in an unbiased way. Our first study with non-irradiated HIP parabionts and allogeneic BALB/c mice focused on the immunogenicity of circulating HIP immune cells. Although HIP immune cells emerged as the a major cell population in BALB/c, we observed a complete lack of immune activation by HIP blood cells, and they successfully engrafted in lymph nodes and spleens. These data suggest that beyond HIP T cells also HIP NK cells and HIP macrophages escape immune recognition in allogeneic recipients. This might be relevant for emerging allogeneic immune cell therapeutics including chimeric antigen receptor (CAR) macrophages,^12^^,^^13^ CAR NK cells,^14^ and regulatory T cell therapeutics.^13^^,^^15^

Our second study with irradiated HIP parabionts served as full-body transplantation model. The irradiation was necessary and effective in avoiding the gatekeeper function of HIP immune cells, which have largely prevented any circulation of BALB/c immune cells in non-irradiated HIP parabionts. Irradiated HIP mice, however, were not able to mount any immune responses against BALB/c cells, which thus could circulate freely and interact with all HIP tissues and organs. Thus, this model allowed the assessment of the total immunogenicity of all HIP organs at the same time. In this study, we relied on very sensitive tests to assess immune activation. The assays used in this study were previously shown to detect subtle immune activation caused by neoantigens that derived from single-nucleotide variations in syngeneic and autologous settings.^16^ In addition, the assays correlated well and were predictive of survival and engraftment outcomes after the injection of neoantigen-harboring cells into syngeneic recipients. The chances for false-negative readouts were therefore considered small. A complete absence of measurable immune activation is only possible if every single organ and tissue that come in contact with allogeneic immune cells fully escape immune recognition. Our data thus suggest that the HIP phenotype in these HIP mice is sufficient to render all cells and organs immunologically invisible for allogeneic immune cells. We have previously shown that Cd47 protects MHC-depleted cells from innate immune cells when expressed above a specific threshold.^17^ This threshold might still be different for different cell types but is below the Cd47 expression levels achieved in the HIP mouse. To confirm that also individual HIP organs can directly be used in allogeneic transplantation, pancreatic HIP islets were exemplarily used. These HIP islets proved to be as functionally active and immune evasive as allogeneic islets that underwent multistep *in vitro* engineering.^3^

Rather than focusing on individual cell types and indications, this study broadly assessed the HIP concept itself. The wide range of conceivable HIP applications open new, so far unexplored cell therapy options for many more immune cell therapeutics and regenerative medicine products.

### Limitations of the study

Parabiosis experiments are limited to short-term follow-up because parabionts in allogeneic settings develop parabiosis intoxication and die between 1 and 2 weeks.^18^ This condition is characterized by wasting, hunched posture, piloerection, anemia, and lymphopenia. However, all our parabionts in the non-irradiated groups survived for the 14-day study period. In the irradiated group, animals were weaker and died earlier. All our animals in the irradiated HIP parabiosis group survived for the 7-day period targeted for this experiment. In mice, early adaptive immune responses peak after 7 days, and thus this study period should have been long enough to study immune recognition. Syngeneic parabionts do not develop parabiosis intoxication and could also survive longer. We designed all non-irradiated experiments for 14 days to be able to compare parameters for syngeneic and allogeneic better. However, besides this time limitation, parabiosis offers unique experimental designs to study unbiased overall immune assessments against either all blood components or all organs at the same time.

## Resource availability

### Lead contact

Further information and requests for resources and reagents should be directed to and will be fulfilled by the lead contact Sonja Schrepfer (Sonja.Schrepfer@sana.com).

### Materials availability

The following materials are restricted: MHC class I and II-deficient mice (B2m and H2-Ab1 double knockout [DKO]; B6.129-*H2-Ab1*^*tm1Gru*^ *B2m*^*tm1Jae*^*)* and HIP mice. Frozen cell suspensions from tissues are not restricted depending on availability and will be available upon request to the lead contact under material transfer agreement with Sana.

### Data and code availability

• No original code was generated in this study.
• All data reported in this paper will be shared by the lead contact upon request.
• Any additional information required to reanalyze the data reported in this paper is available from the lead contact upon request.

## Acknowledgments

We thank in alphabetical order Annabelle Friera, Frank Wells, and Liwen Xiong for *ex vivo* sample retrieval and preparations. Medical Illustration by Justin A. Klein, CMI and Emily Cheng, CMI for Mito Pop.

## Author contributions

X.H. performed all immunobiology experiments, molecular biology, and *in vivo* glucose analysis of diabetic mice and analyzed the data. K.W. performed parabiosis *in vivo* studies. A.G.O. performed histopathology and analyzed the data. C.W. and C.B.C. performed cell suspensions and analyzed immune data. C.G. and C.Y. performed *in vivo* and *in vitro* imaging, respectively, and analyzed the data. A.J.C. evaluated the histopathology and analyzed the data. T.D. evaluated the data, developed and produced the figures, and co-wrote the manuscript with S.S. S.S. conceptualized and designed the experiments, supervised the project, and co-wrote the manuscript with T.D. All authors helped edit the manuscript.

## Declaration of interests

All experiments were conducted by or on behalf of Sana Biotechnology, Inc., and no data from University of California, San Francisco were used. A.J.C. and T.D. performed the work in this manuscript as consultants to Sana Biotechnology, Inc. T.D. owns stock in Sana Biotechnology, Inc. All other authors are employees of and own stock in Sana Biotechnology, Inc. S.S. is inventor on a patent (international application).

## STAR★Methods

### Key resources table

**Table:** 

REAGENT or RESOURCE	SOURCE	IDENTIFIER
**Antibodies**		

anti-CD49b mAb-coated magnetic bead-sorting	Miltenyi Biotec	Cat. no. 130-052-501
EasySep Mouse T Cell Isolation Kit	Stemcell Technologies	Cat. no. 19851
Anti-MHC-I	eBioscience	clone AF6–88.5.5.3
Anti-mouse IgM	Sigma-Aldrich	Cat. no. A9688
Anti-mouse IgG	Sigma-Aldrich	Cat. no. M8642
PerCPeFlour710-labeled anti-MHC class I	eBioscience	clone AF6–88.5.5.3
PerCPeFlour710-labeled mouse IgG2b isotype-matched control antibody	eBioscience	clone eB149/10H5
PerCP-eFlour710-labeled anti-MHC class II	eBioscience	clone M5/114.15.2
PerCP-eFlour710-labeled mouse IgG2a isotype-matched control antibody	eBioscience	clone eBM2a
Alexa Fluor 647-labeled anti-mouse Cd47	BD Biosciences	clone miap301
Alexa Fluor 647-labeled mouse IgG2a isotype-matched control antibody	BD Biosciences	clone R35–95
anti-glucagon	Novus Biologicals	cat. no. NBP2-21803AF647
anti-somatostatin	Novus Biologicals	cat. no. NBP2-99309AF350
anti-insulin	Life Technologies	cat. no. 53-9769-82
PE anti-mouse Cd47	Biolegend	Clone miap301
APC anti-mouse MHC-I	Biolegend	Clone AF6-88.5
Anti-B2m	Abcam	clone EPR21752-214
Anti-GFP	Abcam	Cat. no. ab6556

**Bacterial and virus strains**		

CAG-luciferase LVV	GenTarget	custom order

**Chemicals, peptides, and recombinant proteins**		

poly I:C	Sigma Aldrich	Cat. no. P1530
mouse IL-2	Peprotech	Cat. no. 212-12-1MG
mouse IFN-γ	Peprotech	Cat. no. 315-05-500UG
Mouse TNFα	Peprotech	315-01A-20UG

**Critical commercial assays**		

Total mouse IgM ELISA kit	abcam	Cat. no. ab133047
C-peptide ELISA kit	ThermoFisher	Cat. no. EEL093

**Experimental models: Organisms/strains**		

Female C57BL/6 mice	Jackson Laboratories	cat. no. 000664
Female BALB/c mice	Jackson Laboratories	cat. no. 000651
Female DKO (Abb/B2m) mice	Taconic	custom
Female HIP mice	Taconic	custom

**Software and algorithms**		

Prism 9	GraphPad	
QuPath		

### Experimental model and study participant details

#### Mice

Female C57BL/6 mice (cat. no. 000664, 6–12 weeks), female BALB/c mice (cat. no. 000651, 6–12 weeks) and female C57BL/6 GFP mice (cat. no. 004353, 6–12 weeks) were purchased from the Jackson Laboratories. Female DKO and HIP mice (custom, 6–12 weeks) were generated and purchased from Taconic. Animal experiments were approved by the Explora Biolabs Institutional Animal Care and Use Committee. Animals received humane care and all experiments conformed to the relevant regulatory standards. Mice were housed in 12-h light-dark cycles with humidity between 30 and 70% at ambient temperature of 20–26°C. The animal facility is a specific pathogen-free facility. The number of animals per experimental group is presented in each Figure.

Parabiosis surgeries were performed as previously described.^19^ Briefly, two female and age-matched mice were connected longitudinally from knee to elbow joints, which allowed circulatory systems from two animals to commingle and equilibrate. Some mice were irradiated with 5 Gy prior to parabiosis surgery.

### Method details

#### Mouse NK cell isolation

NK cells were isolated from mice spleen 18 h after poly I:C injection (150 ng poly I:C in 200 μL sterile saline, intraperitoneally, Sigma-Aldrich). After red cell lysis, cells were purified by anti-CD49b mAb-coated magnetic bead-sorting (Miltenyi Biotec, cat. no. 130-052-501) and were stimulated with 1 μg/ml mouse IL-2 (Peprotech, cat. no. 212-12-1MG) overnight for the use as effector cells in killing assays.

#### Single cell suspensions

Mouse spleen, brain, kidney, liver and pancreas was macerated on a 70 μM cell strainer and flushed with RMPI media (Gibco) to obtain a single cell suspension. Heart, lungs, intestine, muscle, colon and skin tissues were cut in pieces and incubated for 30 min at 37°C in RPMI media containing Collagenase XI (2.7 mg/mL, Sigma-Aldrich) and DNAse (0.1 mg/mL, Millipore) before filter the cells through a 70 μM cell strainer. Spleen, kidney, liver, lungs, heart samples were treated with ACK lysing buffer (Gibco) for 5 min at room temperature. Mouse whole blood was treated with ACK lysing buffer only, followed by a washing step with RPMI media.

#### Mouse T cell isolation

Spleen tissues were macerated on a 70 μM cell strainer and flushed with RPMI media (Gibco) to obtain a single cell suspension. T cells were isolated using EasySep Mouse T Cell Isolation Kit (Stemcell Technologies, cat. no. 19851) according to manufactures protocol.

#### Elispot assays

For unidirectional Elispot assays, recipient splenocytes were isolated from spleen 6 or 7 days after cell injection and used as responder cells. Donor cells were mitomycin-treated (50 μg/mL for 30 min) and used as stimulator cells. One hundred thousand stimulator cells were incubated with 1 × 10^6^ recipient responder splenocytes for 24 h and IFN-γ spot frequencies were enumerated using an Elispot plate reader (AID Diagnostika GmbH, Strassburg, Germany). In some groups, effector cells were sorted for GFP-positive or -negative cells or MHC-I-positive (clone AF6–88.5.5.3, eBioscience) and -negative cells. To study immune response after irradiation, mice were irradiated with 5 Gy, followed by cell injection of BALB/c splenocytes on day 1, day 7 or day 14, respectively. Non-irradiated mice served as control.

#### DSA

Sera from recipient mice were de-complemented by heating to 56°C for 30 min. Equal amounts of sera and cell suspensions (5 × 10^6^ mL) were incubated for 45 min at 4°C. Cells were labeled with FITC-conjugated goat anti-mouse IgM (Sigma-Aldrich, cat. no. A9688) and FITC-conjugated goat anti-mouse IgG (Sigma-Aldrich, cat. no. M8642). Analysis was performed by flow cytometry (Attune, Thermo Fisher).

#### ELISA

Plasma was obtained by centrifuging blood for 10 min at 2000 g, and it was utilized to measure total IgM antibodies. Total mouse IgM ELISA kit (abcam, Cambridge, UK, cat. no. ab133047) was used to measure total IgM in mouse serum. Samples were diluted and pipetted according to manufactures instructions. Briefly, standards and samples were added to pre-coated 96-well ELISA plates and incubated for 1 h. After the removal of unbound proteins by washing, anti-IgM antibodies conjugated with horseradish peroxidase, were added. These enzyme-labeled antibodies form complexes with the previously bound compound. The enzyme bound to the immunosorbent is assayed by the addition of a chromogenic substrate, tetramethylbenzidine. Samples were analyzed in a microplate reader (PerkinElmer).

#### Flow cytometry

For the detection of MHC class I/II and Cd47 surface molecules on mouse primary cells, cells were stimulated with 100 ng/mL of mouse IFN-γ (Peprotech, cat. no. 315-05-500UG) and mouse TNFa (Peprotech, cat. no. 315-01A-20UG) for 24 h and labeled with antibodies. For MHC class I: PerCPeFlour710-labeled anti-MHC class I antibody (clone AF6–88.5.5.3, eBioscience) or PerCPeFlour710-labeled mouse IgG2b isotype-matched control antibody (clone eB149/10H5, eBioscience). For MHC class II: PerCP-eFlour710-labeled anti-MHC class II antibody (clone M5/114.15.2, eBioscience) or PerCP-eFlour710-labeled mouse IgG2a isotype-matched control antibody (clone eBM2a, eBioscience). Cd47: Alexa Fluor 647-labeled anti-mouse Cd47 antibody (clone miap301, BD Biosciences) or Alexa Fluor 647-labeled mouse IgG2a isotype-matched control antibody (clone R35–95, BD Biosciences). GFP positive cells were measured in the FITC channel. Viability staining was performed with Zombie NIR Fixable Dye (Biolegend). Cells were analyzed by flow cytometry (Attune, Thermo Fisher) and results were expressed as fold change to isotype-matched control Ig staining or percent positive cells. Histograms were generated with FlowJo v10 and are displayed on a scale that normalizes histogram bins to the mode, or peak, of the histogram.

#### Immune cell killing assays on the XCelligence platform (Live-Dead assays)

Mouse T cell, NK cell, and ADCC live dead assays were analyzed in flow cytometry. 96-well plates were coated either with 0.1% gelatine (Millipore) or with a coating cocktail of collagen (C3867, Sigma), fibronectin (S5171, Sigma) and laminin (A29249, Gibco) for 4 h at room temperature to promote cell adhesion. Forty thousand target cells were plated in 100 μL cell specific media. After 24h, effector cells were added at an effector cell to target cell (E:T) ratio of 1:1. NK cells were stimulated with 1 μg/mL mouse IL-2 (Peprotech). For ADCC, mouse recipient serum was added in addition to unstimulated NK cells. In some groups, effector cells were sorted for GFP positive and negative cells or MHC-I (clone AF6–88.5.5.3, eBioscience) positive and negative cells. As killing control, cells were treated with 2% Triton X-100 (data not shown). Viability staining was performed with Zombie NIR Fixable Dye (Biolegend) for target cells 90h after adding effector cells. Cells were analyzed by flow cytometry (Attune, Thermo Fisher) and results were expressed as percent dead cells.

#### Islet cell isolation and modification

Mouse primary islets were isolated as previously described^20^ and cultured in RPMI 1640 phenol red free, 10% FCS hi, 1% Pen/Strep and 1% Glutamax (mouse islet media, all Gibco). For luciferase transduction, Islet cells were dissociated in single cells using AccuMax (StemCell Technologies) for 10 min at 37°C. CAG-luciferase LVV (custom order, GenTarget) was added at a MOI of 20 and spinfection was performed with the presence of 10 μg/mL protamine sulfate at 300g for 15 min. Cells were replated in U-bottom 96-well plates for islet re-clustering on the belly dancer orbital shaker. After 48h, luciferase expression was confirmed by adding D-luciferin (Promega). Signals were quantified with AMI HT (Spectral Imaging) in maximum photons/cm^2^/sr.

#### *In vivo* BLI experiments

C57BL/6 or BALB/c mice were transplanted with 300 islet clusters intramuscularly into the hindlimb muscle with a 27 G needle on day 0. Mice were monitored on day 0, day 5, day 11, day 13 and subsequently every 4 days until day 29. D-luciferin firefly potassium salt (375 mg/kg, Biosynth) dissolved in sterile PBS (pH 7.4, Gibco, Invitrogen) was injected intraperitoneally (250 μL per mouse) into anesthetized mice. Animals were imaged using the Ami HT (Spectral Instruments Imaging). ROI bioluminescence was quantified in units of maximum photons/cm^2^/sr. The maximum signal from an ROI was measured using Aura software (Spectral Instruments Imaging).

#### Glucose monitoring *in vivo*

To induce diabetes, C57BL/6 or BALB/c mice were injected intraperitoneally with 60 mg/kg streptozotocin (STZ, Sigma Aldrich) for 5 consecutive days. Glucose measurement was performed after 4 h of fasting on day day −5, day −3, day −2, day −1, day 0, day 3, day 7, day 10, day 14, day 21, day 28 with a glucometer (AccuCheck, Roche, Basel, Switzerland). A glucose level above 200 mg/dL was considered as diabetic. For glucose challenge, mice received 2 g/kg glucose solution (Thermo Fisher) intraperitoneally and glucose level was determined 30 min after glucose injection.

#### C-peptide ELISA

C-peptide ELISA kit (ThermoFisher, cat. no. EEL093) was used to measure mouse c-peptide in serum. Samples were diluted and pipetted according to manufactures instructions. Briefly, standards and samples were added to pre-coated 96-well ELISA plates and incubated for 1 h. After the removal of unbound proteins by washing, anti-*c*-peptide antibodies conjugated with horseradish peroxidase, were added. These enzyme-labeled antibodies form complexes with the previously bound c-peptide. The enzyme bound to the immunosorbent is assayed by the addition of a chromogenic substrate, tetramethylbenzidine. Samples were analyzed in a microplate reader (PerkinElmer).

#### Immunofluorescence staining

For islet markers, approximately 20 mL of aggregates were collected into a 1.5 mL Eppendorf tube and spun down. The pellet was resuspended in fixation/permeabilization working solution (cat. no. 00-5523-00, eBioscience) and incubated overnight at 4°C. Cells were washed with permeabilization working buffer (cat. no. 00-5523-00, eBioscience) and stained with 5 μl each of anti-glucagon (cat. no. NBP2-21803AF647, Novus Biologicals), anti-somatostatin (cat. no. NBP2-99309AF350, Novus Biologicals), and anti-insulin (cat. no. 53-9769-82, Life Technologies) for 24 h at 4°C. Cells were washed with permeabilization working buffer, mounted to slides with Prolong Gold (cat. no. P36930, Fisher Scientific), and allowed to dry overnight.

For the immunofluorescence staining of MHC-I and Cd47, 20 mL of aggregates were collected into a 1.5 mL Eppendorf tube and washed with stain buffer (DPBS with 0.1% BSA and 5mM EDTA). Aggregates were stained with 5 μl of PE anti-mouse Cd47 (Biolegend), APC anti-mouse MHC-I (Biolegend) for 45 min on ice and washed with stain buffer. Cells were fixed with BD Cytofix on ice for 30 min (cat. no. 554655, Fisher Scientific), washed with stain buffer, mounted on slides with Prolong Gold with DAPI (cat. no. P36931, Fisher Scientific), and allowed to dry overnight. All slides were then imaged on Leica Thunder Imaging System.

#### Histology

Tissue samples were fixed, processed, and paraffin embedded. Sections were cut to 4 microns for histological analysis. Hematoxylin and Eosin (H&E) staining was done on sections to visualize morphology. Beta-2-Microglobulin (B2m; clone EPR21752-214) and GFP (polyclonal, both Abcam, Cambridge, United Kingdom) antibodies were used to visualize and quantify cell trafficking. Antigen retrieval was performed with an EDTA-based solution. Blocking of non-specific binding was done with 1x Animal Free Blocker (cat. no. SP-5030-250, Vector Laboratories, Newark) in 1% normal goat serum (NGS; Cell Signaling) diluted in TBST (Thermo Fisher). The primary antibody was diluted in blocking solution and applied for 30 min. A secondary anti-rabbit HRP polymer (Leica, Wetzlar, Germany) was used to conjugate DAB staining. Slides were scanned using a 20× objective with a Leica Aperio Versa 200 (Leica). For the QuPath Analysis, the thresholding was determined to identify positive and negative cells and all images were run using the same algorithm. Cell quantification was done with QuPath.

### Quantification and statistical analysis

All data are presented as mean ± standard deviation (SD). Comparisons between two groups were assessed by the Mann-Whitney test. GraphPad Prism 9 was used for all analyses. Animals were randomly assigned to experimental groups. The number of animals per experimental group is presented in each figure.
