# Supplementary material for: The HIP mouse and all of its organs are completely invisible to allogeneic immune cells
Source: iScience. 2024 Nov 28;28(1):111492. doi: 10.1016/j.isci.2024.111492 (PMC11699395; doi:10.1016/j.isci.2024.111492)
Supplement: Document S1. Figures S1 and S2 [file mmc1.pdf]

iScience, Volume *28*

Supplemental information

The HIP mouse and all of its organs

are completely invisible

to allogeneic immune cells

Xiaomeng Hu, Kathy White, Ari G. Olroyd, Chenyan Wang, Carolin B. Caruso, Corie Gattis, Chi Young, Andrew J. Connolly, Tobias Deuse, and Sonja Schrepfer

Supplementary Figures and Legends

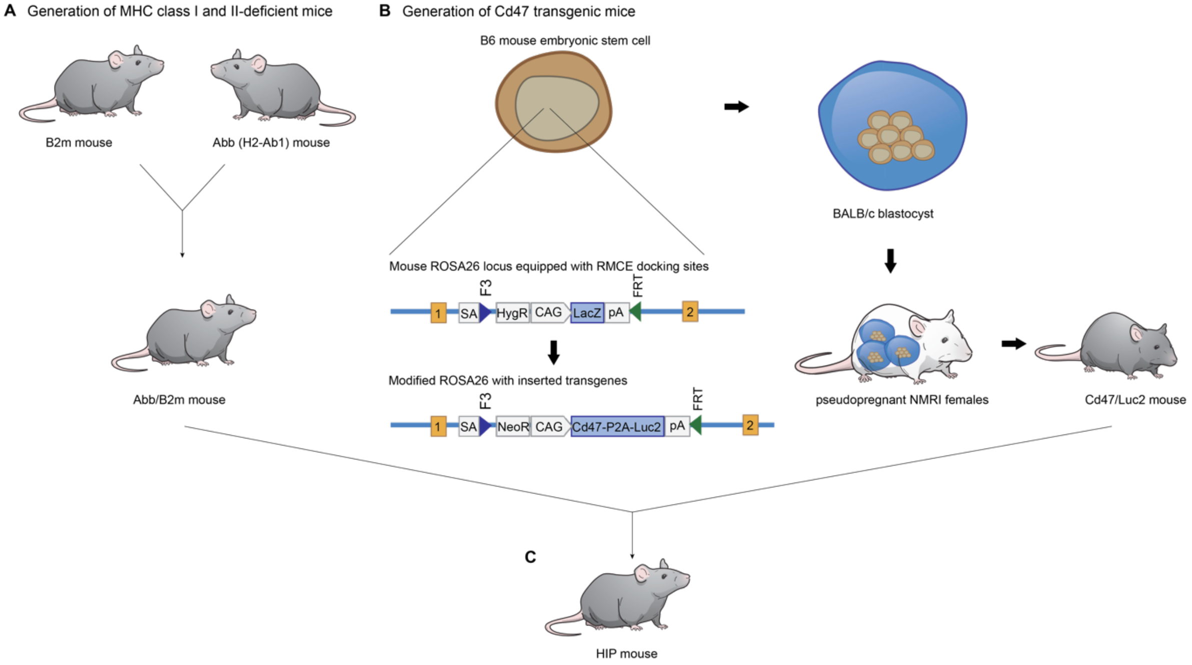

Figure S1: *Engineering of the HIP mice.*
(**A**) MHC class I and II double knockouts of the mouse B2m gene and the mouse H2-Ab1 gene were generated through the mating of B2m mice (MHC class I-deficient) and Abb mice (MHC class II-deficient). (**B**) Cd47 transgenic mice were generated by targeted transgenesis into mouse embryonic stem cells. The recombination-mediated cassette exchange (RMCE) vector was constructed by cloning an F3 site, a CAG promoter cassette, the Cd47-*P2A-Luc2* transgenes, the human growth hormone (hGH) polyadenylation signal, and an FRT site into a plasmid. For targeted transgenesis, the RMCE vector was transfected together with a CAG-*Flpe*-pA expression plasmid into a C57BL/6 embryonic stem cell line equipped with F3 and FRT sites in the *Rosa*26 locus. Recombinant clones were isolated using positive Neomycin resistance selection and expanded. Correctly targeted clones were injected into BALB/c blastocysts and transferred to pseudopregnant NMRI females for chimera generation. (**C**) To obtain HIP mice, female Cd47 transgenic mice were intercrossed with Abb/B2m mice.

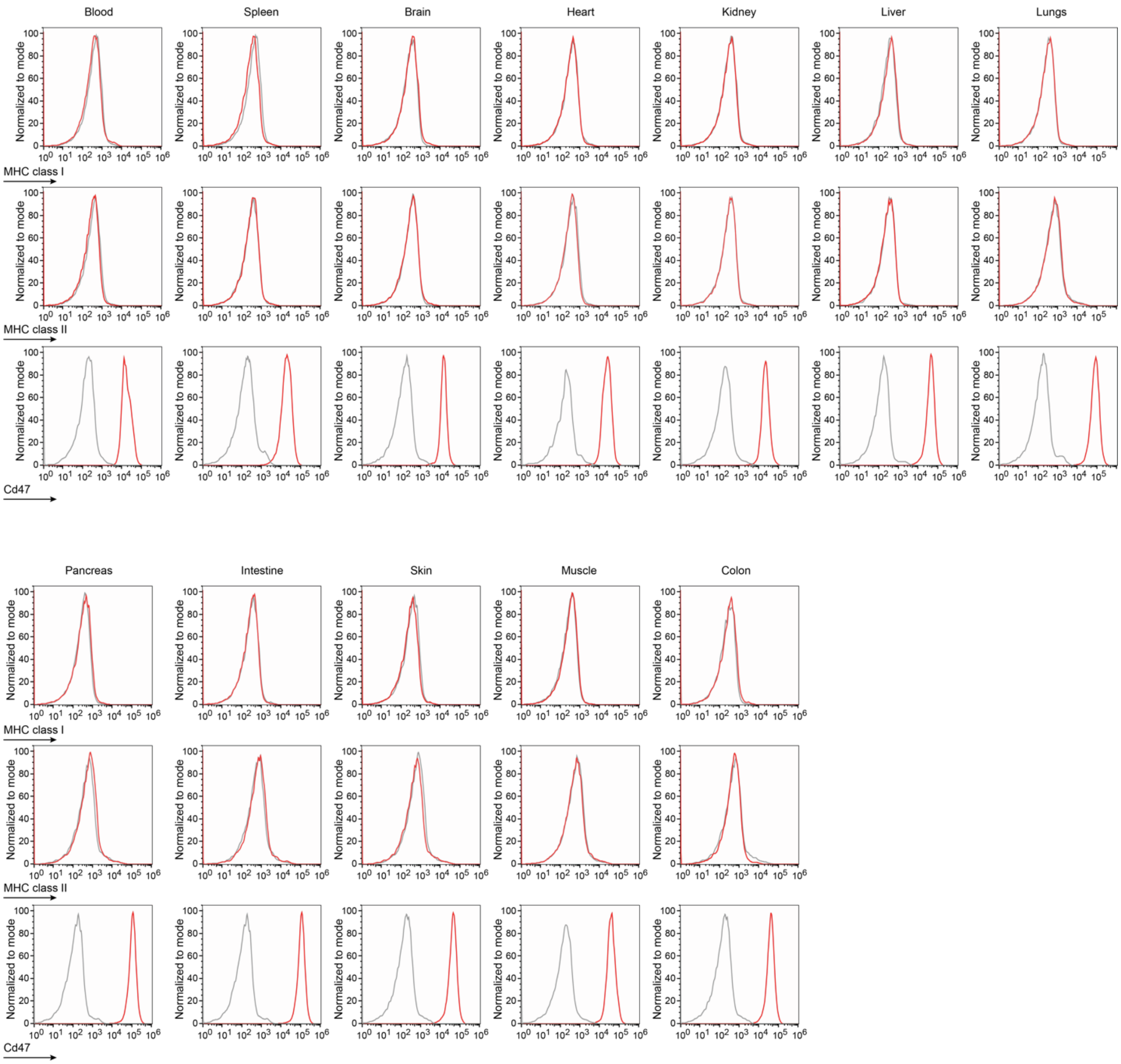

Figure S2: *All cells and organs in the HIP mouse show the HIP phenotype.*

Blood was drawn and multiple organs were recovered from HIP mice and organs were dissociated into single cell suspensions. MHC class I and II and Cd47 expression were assessed by flow cytometry. Histograms show the sample in red and the corresponding isotype control in grey (representative plots of two independent analyses).
